# A Brain Network Processing the Age of Faces

**DOI:** 10.1371/journal.pone.0049451

**Published:** 2012-11-20

**Authors:** György A. Homola, Saad Jbabdi, Christian F. Beckmann, Andreas J. Bartsch

**Affiliations:** 1 Department of Neuroradiology, University of Würzburg, Würzburg, Germany; 2 Centre for Functional MRI of the Brain, University of Oxford, Oxford, United Kingdom; 3 Donders Institute for Brain, Cognition and Behaviour, Radboud University of Nijmegen and MIRA Institute, University of Twente, Nijmegen, The Netherlands; 4 Department of Neuroradiology, University of Heidelberg, Heidelberg, Germany; Institute of Psychology, Chinese Academy of Sciences, China

## Abstract

Age is one of the most salient aspects in faces and of fundamental cognitive and social relevance. Although face processing has been studied extensively, brain regions responsive to age have yet to be localized. Using evocative face morphs and fMRI, we segregate two areas extending beyond the previously established face-sensitive core network, centered on the inferior temporal sulci and angular gyri bilaterally, both of which process changes of facial age. By means of probabilistic tractography, we compare their patterns of functional activation and structural connectivity. The ventral portion of Wernicke's understudied perpendicular association fasciculus is shown to interconnect the two areas, and activation within these clusters is related to the probability of fiber connectivity between them. In addition, post-hoc age-rating competence is found to be associated with high response magnitudes in the left angular gyrus. Our results provide the first evidence that facial age has a distinct representation pattern in the posterior human brain. We propose that particular face-sensitive nodes interact with additional object-unselective quantification modules to obtain individual estimates of facial age. This brain network processing the age of faces differs from the cortical areas that have previously been linked to less developmental but instantly changeable face aspects. Our probabilistic method of associating activations with connectivity patterns reveals an exemplary link that can be used to further study, assess and quantify structure-function relationships.

## Introduction

Processing the age of faces is a crucial cognitive skill. Facial age varies but not in a volatile manner like eye gaze, lip movements or facial expressions. Contrary to other fixed and variant cues [1], the cognitive basis of facial age processing has not yet been investigated by any dedicated neuroimaging experiment. Facial age is depicted by complex features and configurations such as skin texture and distance from average head shape [2]. Localizing a brain network involved with age processing will allow us to determine whether it is primarily up to face-sensitive areas to combine low-level cues into a coherent age percept, whether this is formed by a different system altogether, or whether two or more systems interact with each other. Additionally, facial age processing may also be relevant and applicable to other networks that compute effects of magnitude and passage of time.

Developmentally, age is less salient in faces than gender and ethnicity [3]. In order to augment its processing, we utilize face morphs which provide useful modulations of the age parameter: Attracting visual attention to the optical flow of such changes can be expected to enhance neural responses to every attribute of the morphed object [4], and average response levels measured in virtually all face-selective regions tend to be higher for moving than for static face stimuli [5]–[7]. Morphing is also suited to minimize adaptation while animations facilitate implicit processing without particular cognitive efforts, and video probes in general are increasingly adopted for stimulation in neuroimaging experiments [5], [7]–[16]. Static images from graded morph transitions have already been used as a powerful tool to investigate visual processing of faces and non-face objects [17]–[27] but age has either not been varied or controlled.

**Figure 1:**
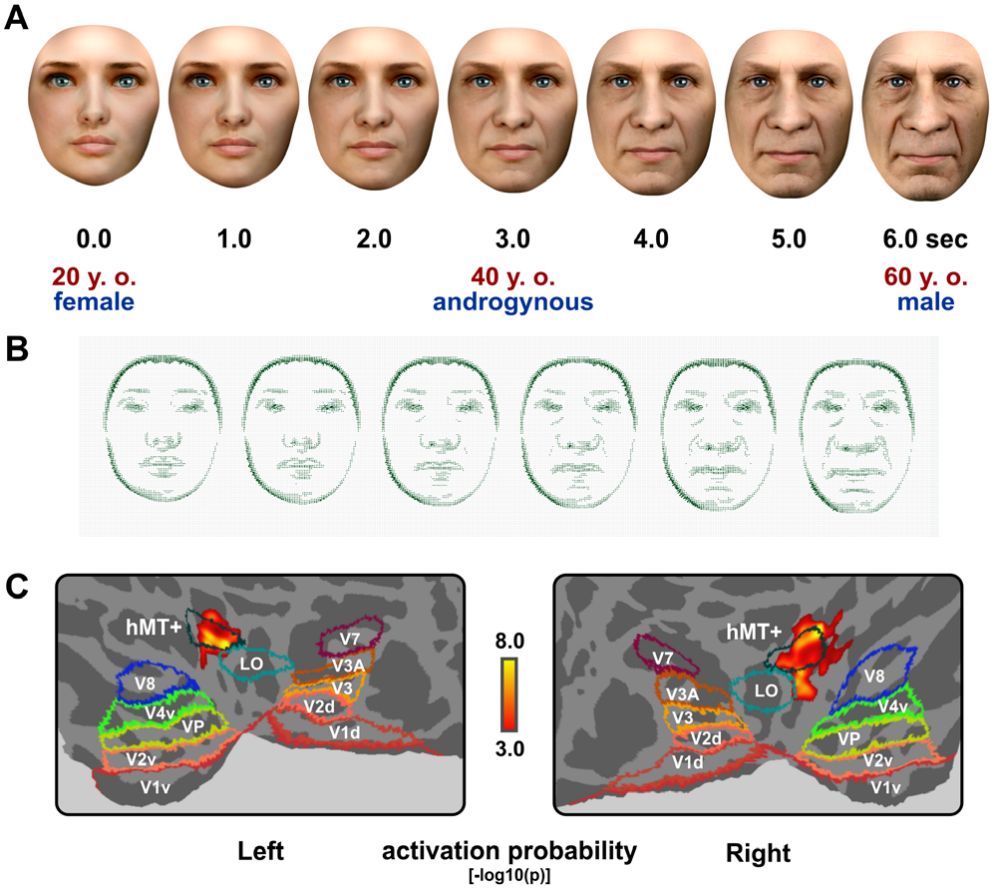
Continuous face morphing, optical flow and associated functional activations. (A) Exemplary keyframes of a video sequence (see Movie S1) morphing a 20 year-old female into a 60 year-old male. Both gradual age and gender changes are illustrated at intervals of 1 second. (B) Line magnitude images of optical flow velocities computed by the Horn-Schunck algorithm. Differential motion/optical flow was quantified as an overall parameter by the sum of flow magnitudes between successive keyframes. (C) Motion-/flow-related activations of hMT+ derived from the group-level analyses (n = 24 subjects, FWER-corrected p<0.05, [−log10 (p)] colorbar) on posterior cortical flat maps of both hemispheres. Additionally, ventral (v) and dorsal (d) visuotopic labels (V1–8, Vp, LO, hMT+) of the SuMS database, transformed from Caret's PALS atlas into FreeSurfer's average surface space, are displayed. Note that according to recent data [112], V4v and V8 are labeled together as hV4 while VP has been labeled V3v.

Here, we generate continuous morphs that introduce independent age and gender changes of face stimuli. These changes are virtual but based on a fully morphable 3D model, similar to [18], [28], and therefore constrained to appear smooth and realistic (Figure 1A, Movie S1). Processing of facial age is compared with gender - instead of identity, attractiveness, or other changeable face aspects - for the following reasons: Gender differentiation normally changes with age and involves changes of similar complexity like aging [29]. By contrast, identity recognition is, within limits, age-invariant [30]. Furthermore, judgments of age and gender need not be affected in prosopagnosia [31]–[33] but cases selectively agnostic for age while preserving gender recognition have not yet been reported. Given that categorical gender varies across androgyny levels [25], both gender as well as age of a face are quantifiable but not instantly changeable. Based on the cognitive models of face processing proposed by Bruce and Young [34] and Ellis [35]–[37], face gender and age have both been conceptualized as ‘visually-directed semantic codes’ [38] and their processing is here compared to each other.

We use functional (fMRI) and diffusion-weighted (DWI) magnetic resonance imaging to investigate the following: *(* ***i)*** Do facial age and gender transitions engage distinct functional networks? *(* ***ii)*** Which of the age-responsive areas are associated with high age-rating competence? ***(iii)*** Is the pattern of functional activation within age-responsive areas related to their connectivity? Using spatial cross-correlations between probability profiles of activation and fiber connectivity, we assess the impact of structural connections on activation patterns. Thereby, we seek to substantiate which fiber pathways transmit age-relevant information of faces. According to our results facial age processing involves distinct brain areas, connected by vertical association pathways. We suggest that face-sensitive regions may interact with quantification modules when age (but not androgyny) is varied across faces.

## Materials and Methods

### Participants

We obtained blood oxygenation level dependent (BOLD) fMRI and DWI scans of 24 healthy, right-handed, white Caucasian volunteers (age range 23 to 34, mean age 26, standard deviation 3 years; 12 females), who all gave written informed consent. The Ethics Committee of the University of Würzburg (Faculty of Medicine) approved the study. Handedness was assessed by a variant of the Edinburgh Handedness Inventory [39], expanded by one eye and foot preference item [40]. Females were scanned between days 5 to 15 of the menstrual cycle, not taking oral contraceptives.

For validation of the tractography results, diffusion-weighted imaging (DWI) data of additional 46 right-handed healthy volunteers (age range 19 to 63, mean age 30, standard deviation 9 years; 25 females) from the database of the Oxford Centre for Functional MRI of the Brain (FMRIB) were analyzed.

### Experimental paradigm

Full-front photographs of 121 unfamiliar, unambiguously gendered faces of white Caucasians (age range 2 to 81, mean age 33, standard deviation 15 years; 60 females age-matched to the males; all free of any make-up and beardless, with eye gaze directed at the viewer, wearing no jewellery or piercings, without tattoos; rated as neutral in their expression on a 6-point visual analogue scale by all participants) were matched by a computerized algorithm [18], [28] to a morphable 3D model consisting of a surface mesh of editable polygons and texture materials using FaceGen Modeller v3.1 (2004, Singular Inversions Inc., Toronto, http://www.facegen.com/) and 3 ds Max 8 (2005, Autodesk Inc., San Rafael, http://www.autodesk.com/). From these models, 120 face morphs were rendered. Half of them contained gender transitions. The mean age difference between start and target face of the 120 morphs was 14.8±11.7 years, ranging from 0 to 46.7 years. Since all face stimuli were generated from a fully morphable 3D model [18], [28], virtual faces between the start and target images appear realistic (see Figure 1A, Movie S1), avoiding grotesque or bizarre distortions for intermediate morphing steps. Face stimuli were presented as shown in Figure 1A. The two subjects selected have given written informed consent (as outlined in the PLoS consent form) to publication of the portrayals derived from their photographs.

Morphing transitions between two faces lasted 6 seconds (see Movie S1), with an additional 1-second still in-between. Face morphs with and without gender transitions (n = 60 each) were arranged in random order, with all morphs across gender proceeding from full-male/-female to the opposite.

Facial age of start and target stimuli was continuously modulated, except for the one-second stills, with varying degrees and age changes being pseudo-randomized according to 10-year intervals. Age changes during morphing were independent of (i.e. orthogonal to) the transitions of gender (Pearson's normalized correlation between the modelled regressors: r = 0.046, p = 0.39) and the psychometric ratings of attractiveness/likeability, which was not significantly modulated (i.e. did not exceed one rating-point difference for all start/target image pairs on a 6-point visual analogue scale). Thereby, morphing eliminates the need for an explicit baseline but nevertheless allows us to separate the effects of age and gender.

**Figure 2:**
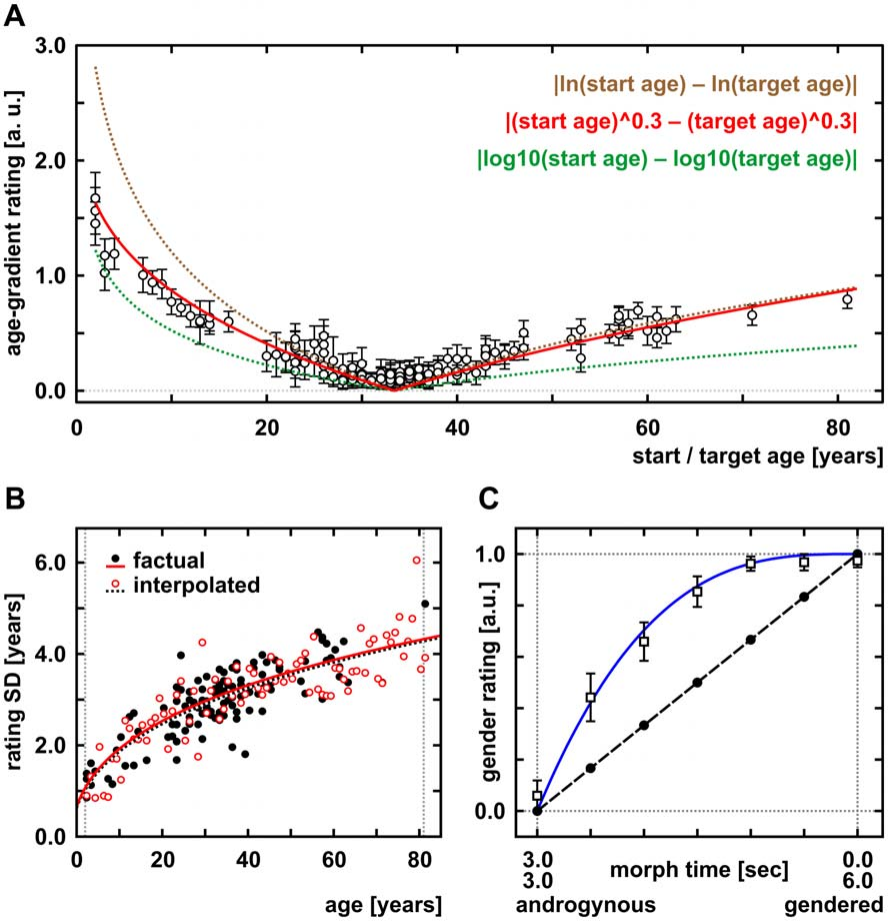
Psychometrics of facial age and gender changes. (A) Facial age difference ratings (magnitude of age-gradients spanned by morphing rated on a 6-point visual analogue scale, maximum scaled to 3.0 arbitrary units [a.u.]) followed Stevens' (∧0.3) better than Weber-Fechner's law (log10) or a natural logarithmic transformation (ln) of start and target age. All face stimuli (n = 121) were morphed to an average-aged male face of 33 years, the morphing sequence was randomly played forwards or backwards for the rating (circles with error bars; n = 24 subjects). (B) Facial aging (x-axis; objective age in [years]) increased the variability of subjective age ratings (y-axis; SD, standard deviation of estimated age in [years] across n = 24 subjects). Rating accuracy of factual (n = 121 stimuli of real faces) and interpolated age (n = 80 intermediate face stimuli from the morphing algorithm; one randomly selected for each annual increment between 2 and 81 years of age) did not differ significantly (p = 0.97). (C) Face gender ratings (on a 6-point visual analogue scale, maximum scaled to 1.0 arbitrary units [a.u.]) along temporal morph continua (n = 60) across faces of clearly different sex. Subjective ratings by (n = 24) subjects (boxes with error bars, blue line) were augmented above linear transition values (dashed line with black dots), reflecting the tendency to apperceptive gender categorization.

**Figure 3:**
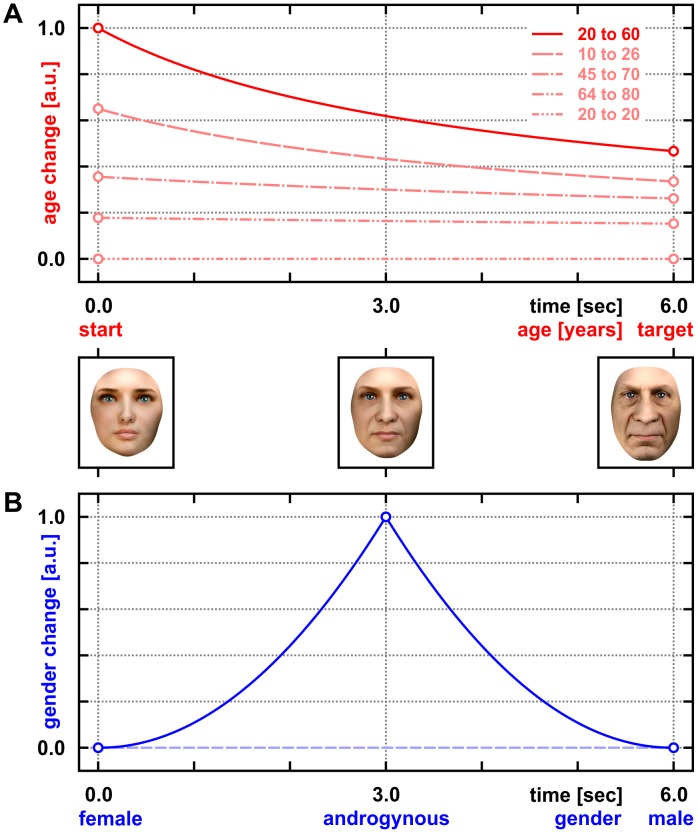
Modeling changes of age and gender during face morphing. Both were time-binned at the video frame rate (24 fps) and scaled to maxima of 1.0 arbitrary units [a.u.]. (A) Differential age change encoded according to Stevens' law of psychophysics (using a power exponent of 0.3; Figure 2A). Note that relative facial aging was up-weighted to initial periods of the example morph (also see Figure 1A; here: solid red line) and, for identical age differences, to younger absolute ages, i.e. aging from 10 to 26 was assumed to provide a stronger stimulus with more visual cues than aging from 64 to 80 years (dashed vs. double-dotted/dashed line). (B) Differential gender change expressed by the first derivative of the function plotted in Figure 2C. Note that peak androgyny was defined as the effective stimulus-of-interest, i.e. the transition of facial gender was emphasized at the center of the morph (see also Figure 1A and Movie S1). Half of the morphs contained no gender transitions, retaining a flat line at zero level to indicate the lack of gender change (dashed line).

Psychophysical changes of age and gender were parametrically modeled according to Steven's power law (cf. Figures 2 and 3). Differential motion/optical flow [9] was controlled for and integrated as a global nuisance variable (Figure 1B,C). Our experimental paradigm simultaneously engaged configural and textural processing, both known to be involved in categorical face processing [41].

### Paradigm presentation

The morphing video was presented at 24 frames per second (fps) using a fMRI-compatible LCD screen, scaled to the maximum resolution of the presentation equipment (640×480 pixels VGA). The paradigm contained the entire set of 120 face morphs between different pairs of start and target faces, separated by 1-second stills, and was presented to the subjects in a single run lasting 14 minutes. Thus, the inter-stimulus interval (ISI) between the offset of a 6-seconds morph and the onset of the next was 1 second, resulting in a stimulus onset asynchrony (SOA) or inter-trial interval (ITI) of 7 seconds with no stimulus repetitions. In order to sustain attention and to monitor compliance, subjects were instructed to press a key with their right index finger whenever the target face appearance of the morphing sequence was anticipated. Speed and accuracy were not emphasized. The explicit task was of no particular interest and only ensured that the participants attended to the paradigm. The associated activations are not reported because they are contaminated by co-activations of executive and motor functioning from key pressing. While watching the face morph of Movie S1, for example, the subject realizes that a young female is changed into an older male, and the characteristics of the target face can be anticipated before it finally appears. This may coincide with the detection of a subjective identity transition although identity virtually changes continuously in a slow but permanent manner during all morphing episodes. Note that age and gender changes were continuously modeled over the entire morphing period while subjects generally pressed the key once in anticipation of the target face, i.e. explicit task performance was sufficiently independent of implicit age and gender processing as we have modeled it. Key-press responses were recorded by a fMRI-compatible keyboard and logged by Cogent 2000 v125 (2003, Wellcome Laboratory of Neurobiology, London, http://www.vislab.ucl.ac.uk/)., Global display luminance was controlled, and constant color channel ratios were maintained. Temporal synchronization between video presentation and scanning was achieved by triggering the start of each fMRI volume externally at a minimum precision of 50 µs using MATLAB R2007a (2007, The MathWorks Inc., Natick, http://www.mathworks.com/).

### MRI and behavioral data acquisition

We acquired fMRI time-series and T1-weighted anatomical images of the (n = 24) healthy volunteers in one session, and whole-brain DWI data and explicit behavioral post-hoc ratings of the same set of subjects in a second session within two weeks. In order to assess the relation between fMRI activations and age rating competence at the second level, age-rating performance was used to discriminate most accurate (n = 5) from average (n = 14) age-raters.

Functional and T1-weighted structural MRI data were acquired on a 3 Tesla TimTrio scanner (Siemens, Erlangen, Germany) using a 12-channel head coil. Whole-brain T2*-weighted BOLD images were recorded by a single-shot 2D gradient-echo EPI sequence with interleaved slice acquisition (TR = 2400 ms, TE = 30 ms, resolution 3×3×4 mm^3^, including 25% interslice gap, 30 sagittal slices of 3.2 mm thickness). After discarding the four initial scans, 350 volumes acquired during visual paradigm presentation were analyzed. In order to unwarp geometric distortions of BOLD EPIs, we used gradient-echo fieldmaps (TR = 500 ms, TE1 = 4.30 ms, ΔTE1/2 = 2.46 ms). In addition, a T1-weighted 3D anatomical image using a MPRAGE sequence (TR = 1560 ms, TE = 2.26 ms, resolution 1×1×1 mm^3^) optimized for segmentation and surface reconstructions and, for basic screening, a T2-weighted 2D axial FLAIR sequence were acquired.

In order to avoid potential DWI signal-loss artefacts [42], we recorded whole-brain diffusion-weighted EPI volumes (60 diffusion directions isotropically distributed on a sphere at b = 1000 s/mm^2^, TR = 9000 ms, TE = 97 ms, resolution 2×2×2.5 mm^3^, including 20% interslice gap, 60 axial slices) and five volumes without diffusion weighting on a 1.5 Tesla Quantum Vision scanner (Siemens, Erlangen, Germany). For unwarping their geometric distortions, gradient-echo fieldmaps matching the DWI protocol were used (TR = 325 ms, TE1 = 4.30 ms, ΔTE1/2 = 4.76 ms).

DWI data of the independent database were acquired on a 1.5 Tesla Sonata scanner (Siemens, Erlangen, Germany) with similar sequence parameters at slightly lower slice thickness (resolution2×2×2 mm^3^, 72 axial slices). Three sets of DWI data were recorded for subsequent averaging to improve the signal-to-noise ratio (total scan time 45 minutes).

### Preprocessing and statistical analysis

All MRI data were processed using FSL 4.1 (http://www.fmrib.ox.ac.uk/fsl/; [43], [44]) and FreeSurfer v4.5.0 (http://surfer.nmr.mgh.harvard.edu/ [45], [46]). First-level fMRI and DWI data were motion- and eddy current-corrected (using MCFLIRT [47] and eddy_correct, respectively), unwarped (using PRELUDE/FUGUE) and brain-extracted (using BET [48]; all part of FSL). First-level fMRI analysis was carried out by applying the General Linear Model (GLM) within FEAT using FILM prewhitening [49], with motion outliers (detected by fsl_motion_outliers) being added as confound regressors. High-pass temporal filtering of the data and the model was set to 100 secs based on the power spectra of the design matrices (estimated by cutoffcalc; all part of FSL). Three main explanatory variables were modeled and controlled: Optical flow, age and gender change. Parametric intensity modulation of their graded stimulus strength is described below, linear modeling of age and gender changes did not explain a significant amount of variance on top of this. Button press responses to anticipated target face appearance were also modeled but of no further interest. In order to capture slight deviations from the model, temporal derivatives of all explanatory variables convolved with FEAT's gamma hemodynamic response function (HRF) were included.

In order to take advantage of surface-based registrations and statistical analyses, FreeSurfer was used for segmentation and surface reconstructions of the structural T1-weighted MRIs. Employing boundary-based registration (using bbregister, part of FreeSurfer, [50]), robust and accurate within-subject cross-modal alignment of functional and anatomical space was achieved. Concatenating this transformation with the surface-based registration to FreeSurfer's spherical average [51], FEAT's first-level contrast-of-parameter estimates (COPEs) and their variance estimates (VARCOPEs) were resampled to the common fsaverage surface. Surface-based spatial smoothing of 5 mm FWHM was applied. At the group level, a mixed-effects (ME) GLM analysis [52] was performed (using mri_glmfit, part of FreeSurfer) identifying vertices in which brain activity was correlated with age, gender and optical flow processing. Second-level thresholding was performed by non-parametric permutation-based cluster mass inference [53]–[57] and included within-contrast correction for multiple comparisons across all vertices of the fsaverage surface. Across the contrasts tested, Bonferroni's correction was applied which further enforced rigorous protection from false-positive detections. Interactions between the main explanatory variables of interest (age and gender change, optical flow) were modeled at the first and assessed at the second level. The volunteers' gender was explicitly modeled at the second level to test for differences between the sexes. Only results with family-wise error rate (FWER) corrected p-values<0.05 are reported, coordinates are given in MNI standard space.

**Figure 4:**
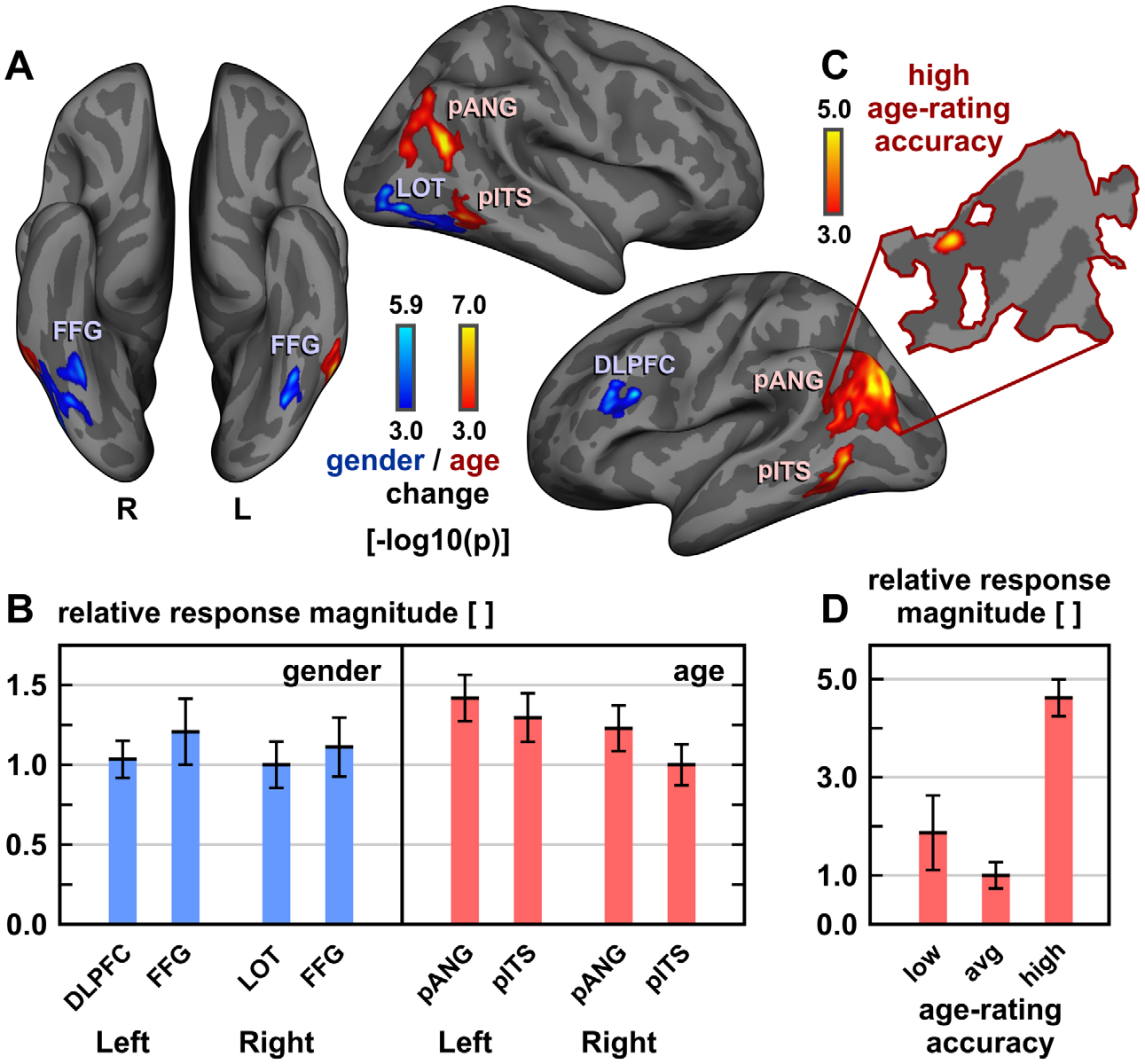
Functional activations associated with changes of facial age and gender. (A) Group-level (n = 24) functional activations^1^ related to age and gender change, respectively. (B) Quantification and between-cluster/-hemisphere comparisons of observed effect sizes evoked by facial age and gender changes across (n = 24) subjects. Individual values of each cluster's mean activation (± error bars across subjects) were normalized to the lowest average of corresponding response magnitudes (as extracted from the first-level analyses). (C) Increased age-related activations^1^ of the most accurate (n = 5) above average age-raters (n = 14). The corresponding cortical flat map is outlined by the borders of the left age-responsive pANG cluster. (D) Relative to average post-hoc raters (avg, n = 14), high explicit age-rating accuracy (upper quintile P80, n = 5) was accompanied by almost five times the response magnitude during implicit age-change processing within left pANG (p<0.001, based on mean individual activation levels of the sub-cluster shown in Figure 4C, as back-projected to native subject space). Activations of lower quintile raters (P20, n = 5) were more variable but not statistically different from the average (P20–80). ^1^Significant activations (FWER-corrected p<0.05) displayed on FreeSurfer's average inflated surface (color bars depict uncorrected activation probabilities [−log10 (p)]). pANG, posterior angular gyrus area; pITS, posterior inferior temporal sulcus; DLPFC, dorsolateral prefrontal cortex; LOT, lateral occipito-temporal area; FFG, fusiform gyrus; orientation labels: L, left; R, right.

Relative response magnitudes were quantified based on individual mean within-cluster contrast-of-parameter estimates (COPEs) normalized to the respective minimum, see Figure 4B,D. Scaled to the peak-to-peak height of the effective regressor and divided by the mean-over-time of the preprocessed (i.e. filtered) EPI time-series from lower-level GLM analyses, mean COPE values are equivalent to mean percentage BOLD signal changes and characterize the observed effect sizes. Given constant scaling for a particular contrast fitted, normalized COPE values translate directly into estimated ratios of the associated signal changes within (but not across) specific contrasts. First-level COPEs from each cluster were tested for hemisphere and sex effects (n = 24; ANOVA, factorial within-/across-subjects design).

**Figure 5:**
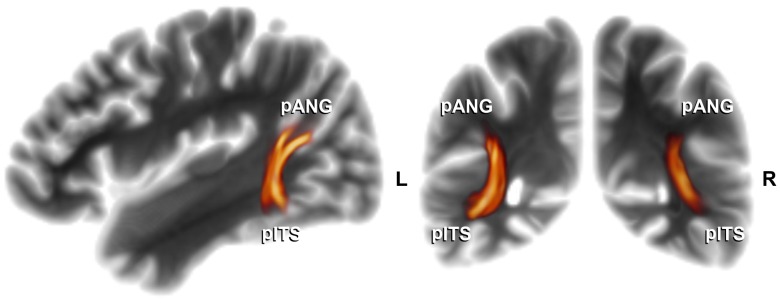
Association pathways subserving facial age processing. Ventral portion of Wernicke's perpendicular fasciculus (WpF) connecting pANG and pITS (average probabilistic path distributions connecting the functional clusters; n = 24, 3D-tract volume rendering thresholded at ≥100 connecting samples passing through each voxel, displayed on sagittal [x = −36 mm] and coronal [y = −54 mm] projection view planes in MNI standard space). pANG, posterior angular gyrus area; pITS, posterior inferior temporal sulcus; orientation labels: L, left; R, right.

DWI data were processed using FMRIB's Diffusion Toolbox (FDT, part of FSL). Up to two fiber orientations were modeled and the corresponding probabilistic distributions of diffusion parameters were built up at each voxel (using bedpostx, part of FDT). Probabilistic modeling of multiple fiber orientations [58] was essential because of crossing fibers in the areas under examination. Subsequently, probabilistic tractography was performed by probtrackx (part of FDT) on the same (n = 24) as well as independent (n = 46) subjects included for replication, to investigate structural connectivity between cortical regions related to either age or gender processing. After transforming functional clusters obtained at the second level back to the individual surface space of the anatomical scans, each cluster mask was defined as a seed with all the others serving as potential targets. Probabilistic streamlines were seeded directly from surface vertices. A total of 10∧4 samples was sent out from each tracking point. Stop and waypoint masking was used to exclude indirect routes. Upon slight spatial smoothing (2 mm FWHM), probabilistic seed-to-target connectivities were then averaged on FreeSurfer's common fsaverage surface. Probabilistic pathways were transformed to MNI space, added and thresholded for visualization (using FSL's non-linear 1 mm MNI template as target space; see 3D-tract volume rendering, thresholded at ≥100 connecting samples passing through each voxel, displayed on sagittal [x = −36 mm] and coronal [y = −54 mm] projection view planes in Figure 5).

Utilizing spatial cross-correlations between functional activation probability values and tractography-based connectivity scores, we examine if the activation pattern of one area that processes facial age is predicted by its intrinsic structural connectivity with another, i.e. evidence for two selected areas directly interacting with each other as connections to the latter determine activations of the former and vice versa. The rationale behind this analysis was that if the spatial profile of a connection between A and B predicts the activation profile in A, then this suggests that the connection between A and B is indeed involved in brain processes producing the activation in A. Because fiber pathways, even when connecting A and B, do not have to participate in the processing, and because functional activations of A and B can be associated with each other in the absence of direct structural connectivity, we don't expect perfect spatial correspondence of functional and connectional probability profiles. But if detectable, significant correspondence of functional and connectivity profiles should emphasize the functional importance of a tract between A and B. Gender processing is again used for within- and across-condition comparison.

**Figure 6:**
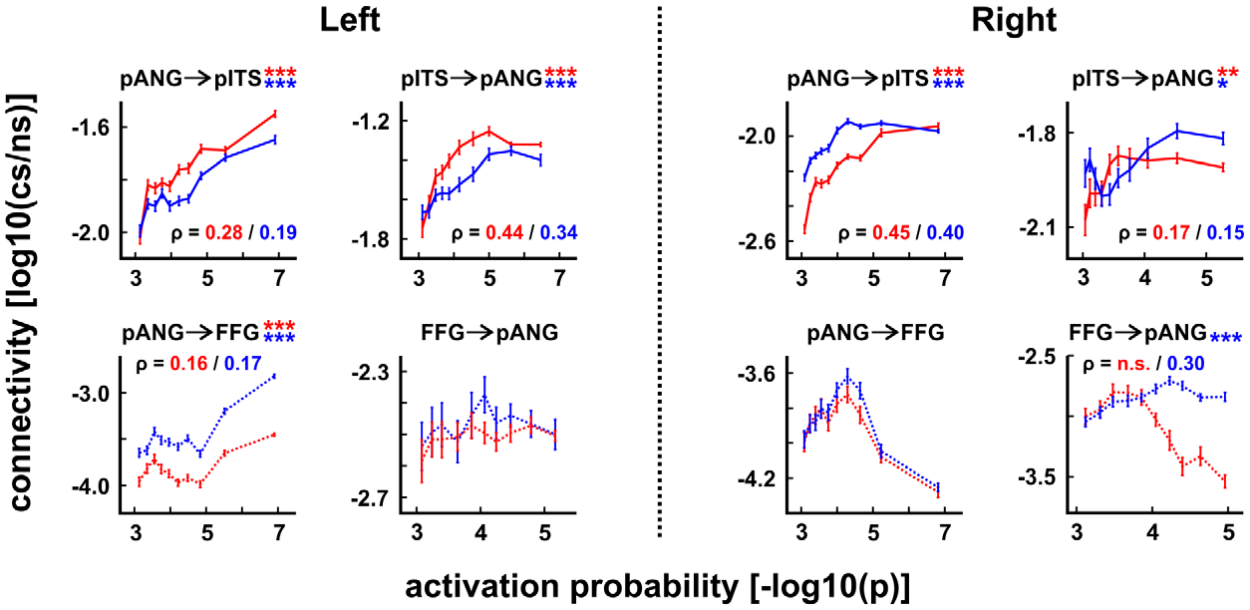
Surface-based cross-correlation of fMRI activation probabilities and structural connectivities. Spatial cross-correlation plots (± SEM)^1^ between activation probabilities ([−log10(p)]) and structural connectivity scores ([log10(cs/ns)], with [(cs/ns)] reflecting ratios of connecting samples to the number of samples sent out from each vertex) for pANG, pITS and FFG (cf. Figure 4A), based on two samples: (n = 24) paired with functional data [red] and (n = 46) independent subjects [blue]. Connectivity ratios tend to get bound earlier at maximum values than corresponding activation probabilities. Positive correlations were slightly stronger for the sample with paired fMRI and DWI data (n = 24) compared to the independent sample from the FMRIB DWI database (n = 46; with Spearman's ρ displayed for the paired/independent sample when significant). However, the latter largely replicate and confirm generalizability of the results. ^1^SEM, standard error of the mean; */**/***: FWER-corrected p<0.05/0.01/0.001. pANG, posterior angular gyrus area; pITS, posterior inferior temporal sulcus; FFG, fusiform gyrus.

Vertex-wise spatial cross-correlations between functional and structural profiles provide a quantitative measure for the association of the two (cf. Figure 6) and were calculated non-parametrically using Spearman's rank correlation coefficient (ρ). All p-values were Bonferroni-corrected for the total number of tests performed. Since lower false-positive activation error probabilities reflect higher activation likelihoods, i.e. higher positive t-values and z-scores, absolute log10 (p)-values were used for correlation.

### Modeling changes of age, gender and optical flow

Since face stimuli underwent continuous temporal changes during the morphing, the explanatory variables of interest were modeled according to their change over time. Age and gender change were time-binned at the video frame-rate (24 fps). Scaling of each regressor was set to a relative maximum of 1. In order to determine accurate stimulus response functions, especially for age and gender, we extensively evaluated our paradigm and the stimuli employed by various psychometric ratings (see Behavioral Results). Thereby, we empirically identified unbiased stimulus response functions for age and gender, later used for modeling in the fMRI analysis.

As illustrated in Figures 2A and 3A, we modeled the age-related changes between start and target faces dependent on their psychophysical age difference. By transforming absolute age using an empirically derived power exponent of 0.3 according to Steven's law, our psychophysical response function accounted for the fact that aging during the first 20 years of life involves more visible changes than from 60 to 80, for example. The power exponent of 0.3 was empirically derived from our psychometric data (see below). According to these psychometric results, absolute facial age is best converted according to Steven's law using 0.3 for exponential transformation to estimate the psychophysical age gradient between start and target face (cf. Figure 2A). For the 6-seconds morphs and based on the psychometric results, the unsigned first derivative is used (cf. Figure 3A). Using the unsigned first derivative is based on the psychometrically tested assumption that the interpolated age of intermediate virtual frames between the start and target face morphed into each other is not differently perceived than the age of real faces (cf. Figure 2B). Thus, our stimulus response function models stronger the age-related activations the higher the psychophysical age gradient between two faces morphed into each other. Based on our psychometric results and in good accordance with previously published data [25], gender was modeled similarly by the unsigned first derivative of linear androgyny levels encoded by Steven's law using 3 for exponential transformation (see below).

Optical flow [59], representing the total amount of motion between successive keyframes of the paradigm, was separately modeled to reduce the amount of unexplained variance which would confound the analysis if all morphing transitions were treated the same. On top of age and gender, optical flow during morphing differs between different pairs of start and target faces. Therefore, the full set of visual stimuli containing the (n = 120) continuous morph sequences displayed in the fMRI paradigm was fed into a customized Simulink V6.6 (2007, The MathWorks Inc., Natick, http://www.mathworks.com/products/simulink/) model estimating optical flow between successive video keyframes by a Horn-Schunck algorithm [60] based on flow velocities using MATLAB's video and image processing blockset with 30 iterations per pair. First, a vector field representing the inter-keyframe motion was extracted, as illustrated by flow magnitude lines in Figure 1B. Then, the vector field was converted to a binary mask. The sum of absolute flow values within that mask described the total amount of motion between successive keyframes of the paradigm and was robustly estimated as a surrogate parameter of overall flow intensity at one-second intervals. Note that optical flow detection on a frame-by-frame basis becomes less robust, i.e. time-binning above 1 fps does not improve the results. Given that all pixels exhibiting optical flow cannot be entered as separate explanatory variables to preserve sufficient degrees-of-freedom for the analysis, and considering that expansions and contractions involved in our face morphing were quite smooth, i.e. cross-correlated, optical flow was estimated at 1 fps and integrated as a nuisance regressor into our paradigm.

## Results

This section is divided into two parts. The first part focuses on the psychometric behavioral data and, based upon these, on modeling the psychophysical changes of the main explanatory variables.

The second part covers the related functional activations, as derived from the same set of (n = 24) subjects, and the structural connectivity between them, i.e. the neuroimaging results of the study.

### Psychometric Results

#### Age rating and the age regressor

For the psychometric assessment, we instructed our volunteers to rate their subjective impression of how much facial age actually changed across morph sequences spanning an age spectrum similar to the original fMRI paradigm. In order to minimize the potential rating bias, the (n = 121) face stimuli were morphed to another average-aged male face of 33 years not contained in the original stimulus set. Therefore, the subjects were familiar with the set of faces used as start and targets in the fMRI video paradigm but not with the particular morphs displayed in the psychometric rating. Single start-to-target morphs were randomly played forwards or backwards for the rating, and the corresponding results are plotted in Figure 2A.

On a 6-point visual analogue scale, subjective age-gradient ratings for the (n = 121) separate face morphs were best encoded according to Stevens' law of psychophysics [61]. Here, the perceived magnitude of age gradients spanned by the morphs was related to the difference between start and target age, both best transformed by a power exponent of 0.3 (cf. Figure 2A). For infinitesimal small bins during continuous morphing, psychophysical age change then corresponds to the unsigned first derivative of absolute facial ages (range: 2 to 81 years) encoded by Steven's law using 0.3 for exponential transformation. Given that it remains unclear to what extent facial age estimates are based on local feature, internal configuration, texture and global shape processing [29], [62]–[64], this was considered the optimal approach to implement and model continuous changes of facial age. Deviations from linear transition values reflect the fact that the subjective perception of facial aging is relatively up-weighted to initial morphing periods and younger absolute ages (Figure 3A). Applied to age changes within individuals, for which only very limited data of unstandardized and scarcely suitable images are available [29], this would reflect a modeling bias towards the earlier development of face shape that prevails over textural changes such as wrinkle formation during later aging [29], or their removal by cosmetic rejuvenation. Across individuals and their shape differences, however, configural processing was not forced into any overt advantage over featural processing (see also the flow magnitude lines in Figure 1B). Both are difficult to completely separate and parameterize for facial age and aging.

In a second rating, the task was to estimate the age of (n = 201) face stills in years. For this purpose, stills of all (n = 121) real faces and of (n = 80) interpolated age models were displayed randomly. In both of these stimulus samples, i.e. the real and interpolated age models, facial aging increased subjective age rating variability across our (n = 24) subjects similar to the group-ratings of young and old faces reported by Ebner [65], see Figure 2B. No significant difference between real and interpolated faces was detected and exponential fitting revealed congruent curves.

#### Age rating competence

Based on the actual distribution of rating errors accumulated over all stills, we trichotomized according to upper and lower quintile cutoffs in order to relate age rating performance to fMRI activations (see below). Because implicit age-change processing (during the fMRI experiment) is rather unlikely to strongly correlate with explicit age-rating accuracy in post-hoc assessments of a limited sample size, this was not more rigorously modeled. The upper quintile of most accurate age-raters (n = 5) was compared to average performers (n = 14). The lower quintile of below-average raters (P20, n = 5) was excluded. Confirmed by their own verbal report, their compliance and motivation was limited at the second session so that age-rating performance of these subjects did not correspond to their actual capacities. This was reflected in disproportionally higher post-hoc rating errors and an increased rating variability (low accuracy ≤P20: 9.1±0.8, average accuracy >P20/<P80: 7.1±0.5 and high accuracy ≥P80: 5.7±0.5 years, as averaged over trials).

#### Gender rating and the gender regressor

In order to investigate the psychophysical processing of gender, especially at intermediate ambiguous levels, (n = 119) sample faces along temporal morph continua across gender from the (n = 60) transsexual morphing sequences were rated by the volunteers on a 6-point visual analogue scale according to their subjective impression of facial gender/androgyny levels. In accordance with previous reports [25], subjective gender levels were augmented above linear transition values reflecting the tendency to apperceptive categorization (Figure 2C). Gender-level ratings of the faces followed Stevens' power law just as age but were best fitted by a power exponent of 3 along the temporal morph continuum which is also in good agreement with the published data [25]. Perceived gender change while morphing thus simply depends on the difference of androgyny levels between time-points. For infinitesimal small bins during continuous morphing towards mid-androgyny between two clearly gendered faces, psychophysical gender change is then described by the unsigned first derivative of absolute androgyny levels (range: 0 to 1), encoded by Steven's law using 3 for exponential transformation (cf. Figure 3B). Note that contrary to previous modeling [25], peak androgyny was defined as the effective stimulus-of-interest because recognition of face gender transitions is particularly emphasized at the center of such morphs, see Figures 1 and 3B. Slight deviations from the center peak were captured by including the temporal derivative (see Material and Methods). Since pre-pubertal faces tend to appear less gendered the younger they are, androgyny peaks while morphing can be shifted to very young faces. Similarly, very old faces, especially when seen without hairstyle, may be liable to a male classification bias. Modeling a temporal derivative accounted for these effects and avoided a bias in testing age-by-gender interactions.

### Neuroimaging Results

#### Functional activations associated with optical flow

Inclusion of optical flow as a nuisance variable enabled us to account for low-level configural and featural changes during face morphing which would otherwise have confounded the analysis. Optical flow was associated with functional activations in the motion-sensitive cortex (hMT+), see Table 1 and Figure 1C, that is known to respond stronger than any other area to radial motion, 2D expansions and contractions of objects [9], [66]–[70]. Functional (hMT+) activations related to optical flow are shown on bilateral flat maps in Figure 1C with additional visuotopic labels [71], [72] of the SuMS [73] database (http://sumsdb.wustl.edu/) to facilitate orientation and to illustrate the spatial correspondence of our clusters with the hMT+ atlas labels.

**Table 1:** Synopsis of functional activations related to age, gender and motion/optical flow.

	hemi	cluster	size	CWP	Max	VtxMax	MNI X, Y, Z			vE/BA	annotation
**age**	left	pANG	2309	0.0001	8.739	142332	−41.0	−74.5	27.0	P*G*/39	inferior parietal
		pITS	526	0.0001	7.307	40331	−54.1	−55.8	−8.5	P*H*/37	inferior temporal
	right	pANG	1177	0.0001	8.465	117820	46.7	−59.3	19.5	P*G*/39	inferior parietal
		pITS	367	0.0004	6.025	5665	54.2	−53.5	−9.4	P*H*/37	inferior temporal
rating*	left	pANG*	32	0.0133	5.045	146872	−42.4	−56.6	25.5	P*G*/39	inferior parietal
**gender**	left	DLPFC	370	0.0005	5.948	29235	−36.9	19.5	22.1	F*D*/46	inf. front. sulcus
		FFG	228	0.0020	5.437	92500	−39.1	−67.9	−17.2	P*H*/37	fusiform
	right	LOT	862	0.0001	5.988	35952	42.4	−77.3	−5.4	O*A*/19	lateral occipital
		FFG	202	0.0021	5.107	28527	36.4	−56.0	−16.4	P*H*/37	fusiform
**motion**	left	hMT+	605	0.0001	8.816	551	−42.6	−79.6	0.1	O*A*/19	middle occipital
	right	hMT+	1204	0.0001	8.554	91197	46.7	−58.9	0.4	P*HO*/19	middle temporal

Clusters significantly activated by changes of facial age, gender and motion/optical flow (FWER-corrected p<0.05 for n = 24 subjects)^1^.

1 hemi, hemisphere; size in [mm^2^], CWP, cluster-wise probability (non-parametric cluster mass inference over the entire surface; [ ]); Max, peak activation probability (absolute log10-maximum of uncorrected p-values: −log10(p); [ ]); VtxMax, vertex of Max on Freesurfer's average surface; MNI, coordinates in MNI standard space [mm]; vE/BA, [113]/[114] area; annotation, anatomical labels; pANG, posterior angular gyrus area (*sub-cluster related to high age-rating competence); pITS, posterior inferior temporal sulcus; DLPFC, dorsolateral prefrontal cortex; LOT, lateral occipitotemporal area; FFG, fusiform gyrus; hMT+, human motion-sensitive MT+ (V5 or MT/MST) area.

#### Functional activations associated with facial age and interactions

Age change-related activations were centered on the posterior inferior temporal sulcus (pITS), lateral to the fusiform gyrus (FFG), and on the posterior angular gyrus area (pANG) of both hemispheres; see Figure 4A and Table 1. Age change, but not the gender condition, was found to be associated with higher mean left-hemispheric activations (p = 0.04, ANOVA, condition-by-hemisphere interaction; Figure 4B). Apart from that, no other significant condition-by-hemisphere interactions, no significant differences between male and female volunteers and no significant positive or negative interactions between the variables-of-interest age, gender and optical flow or their relative response magnitudes, i.e. normalized effect size values, were detected. Note that the fact that we did not detect a gender-by-age interaction may be related to our specific modeling approach and our stimulus set of clearly gendered faces. The latter limited high androgyny levels to morphs with gender transitions and thereby the power to detect such interaction.

#### Age-responsive areas associated with high age-rating competence

Within left pANG, a sub-cluster above the superior temporal sulcus (STS) discriminated the upper quintile (P80, n = 5) of best explicit age raters from average performers (P20–80, n = 14) by higher activations, see Figure 4C and Table 1. Here, superior explicit age-rating competence of upper quintile performers corresponded to an enhanced mean response magnitude in their activation level associated with facial age, which was augmented by more than fourfold relative to average raters (as illustrated in Figure 4D). The lower quintile (P20, n = 5), which was not included in this specific second-level assessment based on the behavioral data, exhibited the highest variability of corresponding relative fMRI response magnitudes (shown by increased error bars in Figure 4D). The more pronounced fMRI response variability of these below-average age raters emphasizes their heterogeneity and presumed underperformance with respect to their actual capacities, which had prompted their exclusion from this part of the analysis (see Behavioral Results above).

#### Functional activations associated with facial gender

Gender change-related activations were detected within FFG bilaterally, see Figure 4A and Table 1, amplifying previous evidence for graded gender responses of the FFG and fusiform face area (FFA) [21], [25]. Notably, increased face androgyny during across-sex morph transitions activated above the more differentiated gender levels (cf. Figure 3B), and not vice versa as for static stimuli [25], illustrating the context dependency of the functional activations. In addition, the right lateral occipitotemporal area (LOT), already implicated by an early PET study [74], and the left dorsolateral prefrontal cortex (DLPFC) were involved (Figure 4A,B and Table 1).

#### Structural connections between age- and gender-related clusters

**Figure 7:**
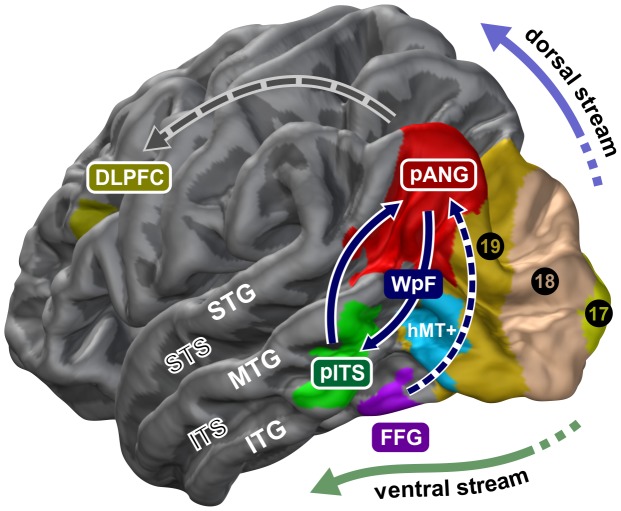
Left-hemispheric nodes of the presumed brain network processing the age of faces. 3D model illustrating how the ventral stream, pITS in particular, may interact via Wernicke's perpendicular fasciculus (WpF) with the posterior magnitude-encoding and approximate number system [98], [99], pANG in particular, to quantify the varying age of faces. FFG exhibits some connectivity to pANG (cf. Figure 6) but is primarily engaged in processing fixed face attributes such as categorical gender (even if continuously changed over variable androgyny levels like in Figure 1A; see also Figures 2C, 3B and 4A). pANG, posterior angular gyrus area; pITS, posterior inferior temporal sulcus; DLPFC, dorsolateral prefrontal cortex; LOT, lateral occipitotemporal area; FFG, fusiform gyrus; 17–19, Brodmann's areas forming three visual tiers; hMT+, human motion-sensitive temporal cortex; ITG/MTG/STG, inferior/middle/superior temporal gyrus; ITS/STS, inferior/superior temporal sulcus.

For each subject, probabilistic tractography was run between all age and gender change-related clusters on individual brain surface reconstructions. An association tract, the ventral portion of Wernicke's perpendicular fasciculus [75], was found to interconnect pANG and pITS (Figure 5). Its almost vertically running fibers connect the posterior inferior parietal lobule, namely the angular gyrus (‘pli courbe’), and the parieto-occipital transition, namely the second parieto-occipital ‘pli de passage’ of Gratiolet [76], with the inferior temporal area [77]. Other cortico-cortical pathways, such as fibers of the superior longitudinal and fronto-occipital fasciculi connecting FFG, pANG and DLPFC (schematically displayed in Figure 7), are not rendered for display and revealed lower connectivities (see log-transformed connectivity scores in Figure 6), except for clusters located very close to each other (e.g., FFG and LOT). Commissure connectivities between clusters related to age or gender processing remained negligible, i.e. less than 0.1‰ of the total number of samples sent out from seed vertices reached the targets (n = 32 pathways of homo-, e.g. right↔left pITS, and heterotopic, e.g. right pANG↔left pITS, commissures extracted).

#### Linking connectivity and activation patterns

The connectivity between functionally defined seed- and target-clusters was quantified by counting how many connecting samples arrived at every vertex of the target (from any vertex of the steed), yielding an index for every target vertex. In order to characterize the extent to which different structural seed-to-target connectivities relate to activation patterns, we then examined the vertex-wise spatial cross-correlations between surface connectivity scores and activation probabilities, i.e. the above connectivity index was correlated (plotted) against the activation probability of the target vertices in Figure 6 (see Materials and Methods). This is based on the hypothesis that if there is a direct structural connection between a pair of functional regions, where this connection itself is involved in the cerebral processing, then locations of highest fMRI activation probability within the regions should be close to the highest probability of structural connectivity between them, i.e. the patterns of functional activations and anatomical connectivity should be positively correlated. We tested this hypothesis on all clusters responsive to age and gender change.

At the group level, by far most consistent spatial cross-correlations between activation probabilities and average connectivity scores were detected for pANG and pITS (cf. Figure 6; results exclusively shown for association pathways of Wernicke's perpendicular fasciculus between pANG, pITS and FFG). Thus, for pANG, lowest type I activation error probabilities were in proximity to high pITS connectivity, indicating pITS' ability to directly recruit pANG for age processing and vice versa. In addition to pANG and pITS *within* the age-change condition of both hemispheres (Figures 4 and 6), significant but lower positive vertex-wise spatial cross-correlations of activation probabilities and connectivity values were only found in the right hemisphere for the pANG and LOT cluster (0.10≤ρ≤0.18 for pANG↔LOT; not shown). Furthermore, activations of the pANG cluster revealed potentially relevant associations with connections to FFG, especially on the left, but just on this tractography seeding end (i.e. FFG activations were *not* consistently related to their pANG connectivity; see spatial cross-correlation plots in Figure 6), and pANG's overall connectivity to the FFG cluster remained comparatively low. These connections, which were originally discovered by Wernicke [78] based on anatomical examination of monkey brains [79] and later demonstrated to form part of the stratum verticale convexitatis in men [80], correspond to fibers of Wernicke's perpendicular fasciculus between the angular and fusiform gyri [81]. Commissure connectivities (n = 32 homo- and heterotopic pathways; see above) were also tested but revealed no significant vertex-wise spatial cross-correlations with corresponding activation probabilities. Because cluster size and distance effects are hard to disentangle from meaningful inter-hemispheric differences, we did not test for differences between surface-based spatial cross-correlations of fMRI activation probabilities and structural connectivities across the two hemispheres.

## Discussion

Our investigation provides the first description of a distinct brain network associated with processing the age of faces and its underlying structural connectivity. Although facial age is of high social relevance and has been shown to influence medial prefrontal activations when presumed personality characteristics are rated [82], the neural basis of facial age processing itself has not previously been identified. According to our data, its primary cortical components – the areas around pITS and pANG – are separate from those processing categorical gender while face-selective FFA activations have recently been shown to be primarily predicted by inferotemporal connectivity of the FFG [16]. Notably, the age-processing network, i.e. pITS *together with* pANG, also expands the proposed core system representing other variable face aspects (such as eye gaze; unvaried and directed at the viewer in our fMRI study) which are ready to change instantly under attentional, emotional or volitional instead of ontogenetic control [1], [83]. It may also augment the extended system, as previously conceptualized, which has been suggested to mediate spatially directed attention and recognition of emotions in faces, for example [1].

### Structural within-network connectivity

Wernicke's perpendicular fasciculus (WpF) has been recognized as a separate cerebral association tract but so far largely escaped further description and attention [84]. Only its more posterior occipital portion has been implied as part of a disconnection syndrome potentially underlying cases of pure alexia [85]. Compared to other association tracts, it runs in a peculiar vertical instead of horizontal trajectory which enables it to interconnect ventral and dorsal parallel pathways [86], see Figures 5 and 7. Despite the known limitations and inaccuracies of DWI-based tractography [87], our main finding that brain activity spatially correlates with connectivity between pITS and pANG substantiates our claim that the structural connection of these two cortical areas by WpF's ventral portion is of real functional importance *in vivo*. Specifically, our results suggest that this association pathway may be linked to facial age processing (see below).

### Methodological Limitations

Given that tractography cannot reveal directions of information processing, our data do not ensure that it is pITS that recruits pANG and not vice versa. Continuous morphing, despite its strength of presumably augmenting change-sensitive neural responses and supporting our explicit psychophysiological model, precludes reliable detection of temporal delay differences between pITS and pANG. Thus, we acknowledge that these clusters may influence each other reciprocally, as schematically indicated in Figure 7, a fact that is generally assumed for most cortical areas connected by association tracts.

Due to the inherent smoothness of fMRI data, which is further pronounced on the average surface, and because connectivity probabilities tend to increase the closer seed and target are located, association of probabilistic connectivity and activation probabilities for extremely short association fibers and cortical regions located very near to each other (e.g., right LOT and pITS *across* the age and gender condition) may be inflated. In fact, at a liberal threshold (p = 0.05, uncorrected for multiple comparisons) the right age-related pITS activation extends into the ipsilateral gender-related LOT cluster, and nearby parts of these two clusters are also vigorously interconnected. This suggests that right LOT, to some extent, may participate in age processing. Similarly, connectivity of gender-responsive FFG to nearby pITS (not shown) and pANG (Figure 6) may be relevant for facial age processing. In having identified potential key components of these circuitries, our study may serve as a precursor for future studies of effective functional connectivity between such sets of areas, e.g. by using dynamic causal modeling (DCM). Interestingly, apparent face gender is normally fixed for a given individual (except in early developmental stages and transsexuals): Changes of face gender are virtually always and inevitably associated with changes of personal identity. Consistent with it and the presented results, both gender and identity processing have been strongly associated to FFG/FFA [19], [21], [25], [88]–[93]. The fact that our data on the processing of face gender (which also changed continuously in our experiment) largely replicates previous results obtained by static stimuli [21], [25], [74], [94] and that neither our age nor gender condition involved the right anterior STS (which has been shown to exclusively respond to moving faces) [7], [11] further strengthens the conclusion that we have indeed detected a brain network processing the age of faces and not other unstable features (such as the movement within the faces) or subliminal changes of identity. This corroboration is important, since people do not normally age in front of our eyes, and adaptation experiments have indicated the potential recruitment of more widely distributed brain regions [21] and the area around the STS even in identity processing [95]. However, the latter finding is not supported by other data [96] and would, contrary to our findings, also be expected to affect our gender condition.

### Interpretation

A unique combination of fMRI and diffusion tractography measurements enabled us not only to track anatomical connections between peak activations but also to uncover significant spatial cross-correlations between functional activations on the one hand and structural connectivity probabilities on the other. The demonstration of such associations between functional and probabilistic connectivity measures, which has only recently been highlighted by a different hypothesis and approach [16], [97], may further assist the understanding of relations between fMRI activations and cerebral connectivity. For example, this type of association in the localization of function and structure can substantiate evidence for fiber tracts being directly involved in transmitting condition-relevant information which has not previously been proposed or analyzed. Our data suggest that age-relevant information may be transmitted between pITS and pANG by the anterior division of Wernicke's perpendicular fasciculus to which no unambiguous function has yet been attributed.

Our results allow us to propose the first coherent model (illustrated in Figure 7) for how the ventral visual stream may interact with the angular gyrus area to process different facial ages. Changing within as well as across individuals, age is automatically quantified and attributed particularly to faces, i.e. more precisely than for any other object. We propose that facial age is represented in terms of growing quantities and contrastable numerical magnitudes. By utilizing the ventral part of Wernicke's perpendicular fasciculus, a largely understudied association tract, age-responsive pITS gains access to pANG, posterior processing core of a common quantification network outlined in a theory of magnitude (ATOM) [98] and part of an approximate number system (ANS) [99]. Neither pITS nor pANG are likely to process facial age exclusively, and both areas are certainly not only devoted to decode the age of faces, yet our findings indicate their spatially coherent involvement in its implicit processing across subjects. Sparse data from brain-lesioned patients [100] indicate that the posterior right brain may be crucial for (ap-)perceptive component of age processing. Increased left-hemispheric responses (shown in Figure 4A,B) and higher activation levels of most accurate raters above the superior temporal sulcus within left pANG only (cf. Figure 4C,D), on the other hand, correspond to a known importance of the left angular gyrus for abstract number representations, quantized discrete decoding, numerical comparisons and operations [101], [102], as well as high mental calculation abilities [103]. These findings do not disambiguate which lower-level brain regions and face features (e.g., wrinkle quantity, skin texture, or head cast with different developmental face-to-skull proportions) contribute to the encoding of facial age. Nevertheless, they may suggest that the age-responsive network assembles and eventually integrates these inputs into comparable and estimable magnitudes particularly within pANG, by different contributions from the right and left hemisphere. Contrary to the static and dynamic processing of other face object categorizations and discriminations [7], [104], average responses related to facial age were more pronounced and robust in the left hemisphere, and age-rating performance was also associated with left brain functioning of pANG.

Considering the extraordinary relevance of age judgements for the interpersonal domain, e.g. to establish peer communication, attractiveness and even empathy, the cognitive processing of facial age and aging reflects an intrinsic core capacity of the human “social brain”. Its functions have also been related to the temporo-parietal junction. Attractiveness, which has not been modulated in our study, is influenced by facial age [2], [105]. It has been, among other brain areas, associated with the STS. However, activation peaks reported for attractiveness judgements [106] were located superior to those of pITS and more anterior to those of pANG (see Table 1). Obviously, faces of the same age are differently attractive, and attractiveness per se has not been shown to evoke unequivocal activations [107]. Furthermore, the brain network proposed by us to process facial age clearly differs from the largely reward-related systems that have been implied in association with beauty [107]–[110].

### Outlook and Conclusions

Future investigations and lesion studies are required to further elucidate cognitive age processing. Our analysis may be broadened by other approaches examining distributed patterns of neural age-encoding in their selectiveness and specificity but this would have been beyond the scope of this study. More elaborate insights can be anticipated investigating age discrimination upon face inversion, processing the age of non-face objects, adaptation to age, own- vs. other-age effects including associated visual processing strategies and their potential center-periphery bias, cross-modal integration of age information, age processing in the blind, dissociation of non-abstract and numerical age representations, and the development of age-recognition expertise. Even though our study highlights pANG as one key component for age processing, its precise role in this context is still speculative and needs further investigation. Our model, illustrated in Figure 7, gives rise to interesting hypotheses: One testable prediction would be that disruption of left pANG activity using transcranial magnetic stimulation (TMS), for example, should impair numerical age but not gender judgements, and that brain lesion-symptom mapping can eventually dissociate the two. On the other hand, our model of separate brain networks processing age and gender (cf. Figure 7) would be falsified if dissociations of age from gender agnosia cannot be confirmed. Consistent with our proposed model of segregated neural systems for gender and age processing, Bruyer & Schweich describe a patient with prosopagnosia secondary to a right temporo-occipital brain haemorrhage (most likely affecting right LOFA +/− FFG according to their description) who exhibited deficient gender categorization but in whom age classification was preserved [38]. Another assumption would be that activity of at least parts of the network processing the age of faces is incremental with low-level cues, such as wrinkle formation and head proportions, with more cues giving rise to higher activation levels in these areas. Such low-level cues determining facial age perception may be modulated con- and divergently. Finally, pANG (but not pITS?) may be involved in the processing of age and aging for non-face stimuli, like other body parts or inanimated objects.

The notion of a distributed neural core system processing fixed attributes vs. changeable aspects of faces [1] parallels the traditional logic of distinguishing essential from non-essential object properties. In this regard, facial age may be considered an auxiliary appearance, conveying non-symbolic, abstract and social information. As is the case with gender and identity, age does not need to be constantly ascertained in another's face and is not reproduced or “mirrored“ by the perceiver. Therefore, its regular processing presumably relies less on permanent monitoring required to follow eye gaze, lip-speech or facial expressions [83] for which predictable motion trajectories may be anticipated [111]. Our results can be interpreted to discover a genuine set of the face-processing ensemble: the posterior inferior sulcus within the extrastriate system of visual face analysis that interacts via Wernicke's perpendicular fasciculus with extended modules of the angular gyrus area to represent the age of faces.

## Supporting Information

Movie S1 **Exemplary video sequence morphing a 20 year-old female into a 60 year-old male.** The exemplary morph video (Movie 1) is also available in Windows AVI and Apple QuickTime format for download here: http://www.neuroradiologie.uk-wuerzburg.de/facemorph/.
(MOV)
